# Pathologic Findings From Conization as Predictors of Residual Disease in Stage IA2 Cervical Cancer: Implications for Fertility-Preserving Surgery With Sentinel Lymph Node Biopsy

**DOI:** 10.7759/cureus.85651

**Published:** 2025-06-09

**Authors:** Shinichi Togami, Nozomi Furuzono, Takuto Isomichi, Mai Kuribayashi, Mika Fukuda, Hiroaki Kobayashi

**Affiliations:** 1 Department of Obstetrics and Gynecology, Faculty of Medicine, Kagoshima University, Kagoshima, JPN

**Keywords:** cervical cancer, conization, fertility preservation, sentinel lymph node biopsy, stage ia2

## Abstract

Introduction: Conization is a standard fertility-preserving treatment for stage IA1 cervical cancer; however, its role in stage IA2 disease remains controversial due to limited supporting evidence. This study aimed to evaluate the feasibility of conization with or without sentinel lymph node (SLN) biopsy as a fertility-sparing strategy in patients with stage IA2 cervical cancer.

Methods: We retrospectively analyzed 20 patients diagnosed with stage IA2 cervical cancer based on conization pathology who subsequently underwent modified radical or radical hysterectomy or trachelectomy with SLN biopsy at Kagoshima University Hospital between March 2014 and December 2023. SLN mapping was performed using a hybrid technique combining technetium-99m and indocyanine green. Residual tumor presence in hysterectomy specimens and associated clinicopathologic factors were evaluated.

Results: The median age was 45 years. Histologic subtypes included squamous cell carcinoma (75%) and adenocarcinoma (25%). SLN mapping was successfully performed bilaterally in all patients (100%), with no SLN metastases detected. Residual tumor was identified in eight patients (40%). Positive endocervical curettage (ECC) was significantly associated with residual tumor (OR = 3.38, 95% CI: 1.31-8.76, p = 0.035). Notably, no residual tumor was observed in patients with both negative ECC and negative endocervical margins. Among the five patients who underwent trachelectomy, all preserved their fertility potential during the follow-up period, and no recurrences were observed during a median follow-up of 45 months.

Conclusion: In patients with stage IA2 cervical cancer, positive ECC and adenocarcinoma histology were significantly associated with residual tumor in the final hysterectomy specimen. Importantly, no residual tumor was observed in patients with both negative ECC and negative endocervical margin, particularly among those with squamous cell carcinoma. These findings suggest that for highly selected patients, especially those with squamous histology, conization ± SLN biopsy may represent a safe and less invasive fertility-preserving alternative to trachelectomy. Further prospective studies are needed to confirm oncologic safety and define appropriate selection criteria, particularly for cases of adenocarcinoma.

## Introduction

Cervical cancer remains one of the most common gynecologic malignancies among women of reproductive age worldwide [1]. Recent advances in early detection and the widespread implementation of cervical cancer screening programs have contributed to a trend toward earlier diagnosis, particularly among younger women. In parallel, the introduction of HPV vaccination has significantly reduced the burden of premalignant cervical disease, which may lead to long-term changes in cervical cancer incidence and age distribution [2]. As a result, fertility preservation has become an increasingly important consideration in the management of early-stage cervical cancer, particularly among women of reproductive age.

Trachelectomy has emerged as a fertility-sparing surgical option for select patients with early-stage cervical cancer. However, while oncologically effective, trachelectomy is associated with potential reproductive and obstetric risks. These include cervical stenosis, second-trimester miscarriage, and preterm birth, which may impact long-term fertility and perinatal outcomes.

To address these challenges, this study aims to evaluate the feasibility and safety of trachelectomy combined with sentinel lymph node (SLN) biopsy as a less invasive, fertility-preserving strategy in patients with stage IA2 cervical cancer. Our primary research question is whether this approach can achieve acceptable oncologic and reproductive outcomes while minimizing surgical morbidity.

For patients with stage IA1 cervical cancer, defined as stromal invasion of ≤3 mm and horizontal spread of <7 mm, conization is an established standard fertility-sparing treatment. When surgical margins are negative and lymphovascular space invasion (LVSI) is absent, conization is considered oncologically safe and has been associated with favorable fertility outcomes. In contrast, patients with stage IA2 disease, characterized by stromal invasion of >3 mm and ≤5 mm with horizontal spread of <7 mm, are typically recommended to undergo more extensive surgical intervention. According to both the Japan Society of Gynecologic Oncology and international guidelines [3,4], patients with stage IA2 disease who desire fertility preservation are advised to undergo a modified or radical trachelectomy in combination with pelvic lymphadenectomy or SLN biopsy.

Radical trachelectomy, performed via vaginal, abdominal, or minimally invasive approaches, has demonstrated satisfactory oncologic and fertility-sparing outcomes in appropriately selected patients [5]. However, despite its oncologic efficacy, radical trachelectomy is associated with significant reproductive and obstetric complications, including cervical stenosis, infertility, second-trimester miscarriage, and preterm delivery [5]. These complications can pose serious concerns for young women who wish to preserve their fertility.

Given these issues, interest is growing in less invasive alternatives to trachelectomy for patients with stage IA2 disease, particularly in those with small-volume tumors and favorable histopathological features. Conization, which is already a standard treatment for stage IA1 disease, has been reported in selected studies to be a potentially feasible option for some IA2 patients. When negative surgical margins are achieved and both LVSI and lymph node metastasis are absent, conization may offer a safe and less invasive alternative to trachelectomy [6].

However, clinical evidence regarding the oncologic safety and efficacy of conization alone for stage IA2 cervical cancer is extremely limited. Existing reports are generally based on small sample sizes, and there is considerable heterogeneity in patient selection criteria, surgical techniques, and follow-up protocols [7]. Furthermore, there is currently no established consensus on the specific subset of IA2 patients for whom conization might be considered oncologically acceptable. This uncertainty contributes to hesitancy among clinicians to adopt conization as a definitive treatment strategy for IA2 disease.

In addition, the rarity of stage IA2 cervical cancer itself presents a challenge for conducting large-scale, high-quality studies. IA2 comprises only a small proportion of early-stage cervical cancer cases, and much of the available evidence is derived from multicenter registries or retrospective analyses. Moreover, most of these studies have been conducted in Western populations, and differences in patient demographics, tumor characteristics, healthcare systems, and surgical practices may limit the generalizability of these findings to Japanese patients.

In Japan, radical trachelectomy remains the standard fertility-preserving surgical option for patients with stage IA2 cervical cancer. However, domestic data on the use of conization alone for this indication are extremely limited. To date, there have been very few peer-reviewed studies published by Japanese institutions examining the clinical outcomes of conization in IA2 patients. Consequently, Japanese clinicians lack sufficient evidence from local cohorts to guide patient selection and counseling in this context.

Against this background, the present study aimed to evaluate the feasibility of conization as an initial surgical approach in patients with stage IA2 cervical cancer. We conducted a retrospective analysis of cases treated with conization and assessed clinicopathologic features and oncologic outcomes, including recurrence and survival. This study sought to investigate whether conization may represent a reasonable alternative to trachelectomy in selected patients with stage IA2 disease.

## Materials and methods

Population

This study included patients who underwent surgery for early-stage cervical cancer at the Department of Obstetrics and Gynecology, Kagoshima University Hospital, between March 2014 and December 2023. Eligible patients were those who were diagnosed with stage IA2 cervical cancer based on pathological assessment of conization specimens. Histological subtypes included squamous cell carcinoma, adenocarcinoma, and adenosquamous carcinoma. Patients with synchronous malignancies or those in whom SLN biopsy was not performed were excluded.

Intervention

Surgical procedures consisted of either modified radical or radical hysterectomy or trachelectomy, combined with SLN biopsy. The choice of surgical method was primarily based on patient preference following detailed counseling on the risks and benefits of each approach. All procedures were performed via either laparotomy or laparoscopy by four board-certified gynecologic oncologists. Laparoscopic surgeries were carried out by two surgeons certified by the Japan Society of Gynecologic and Obstetric Endoscopy and Minimally Invasive Therapy.

This study was retrospective in nature, and therefore, no formal sample size calculation or power analysis was conducted. All eligible patients with stage IA2 cervical cancer who met the inclusion criteria during the study period were included for analysis.

SLN detection protocol

The details of SLN identification have been described in our previous study [8,9]. SLN mapping was conducted using a hybrid method that combined technetium-99m-labeled tin colloid (RI method) and indocyanine green (ICG). On the day prior to surgery, 111 MBq of technetium-99m phytate was injected into the uterine cervix. Lymphoscintigraphy and SPECT-CT (single photon emission computed tomography-computed tomography) were performed to estimate SLN locations. Immediately before surgery, diluted ICG (10×) was injected into the cervix. During surgery, SLNs were visualized using a near-infrared fluorescence imaging system and confirmed as "hot nodes" using a gamma probe.

Intraoperative rapid examination was used to evaluate SLN metastasis. If no metastases were found, systematic pelvic lymphadenectomy was omitted. In cases with SLN metastasis, systematic pelvic lymphadenectomy was performed.

Outcome and statistical analysis

The primary outcome was the feasibility and oncologic safety of SLN biopsy-based management in patients with IA2 cervical cancer. Categorical variables were compared using the chi-squared test or Fisher's exact test, and continuous variables were analyzed using the Wilcoxon rank-sum test. A p-value < 0.05 was considered statistically significant. A multivariate logistic regression analysis was conducted using four clinicopathological variables that were considered to have a direct association with residual tumor in the uterus following conization. These variables included histologic type, endocervical margin status, ectocervical margin status, and endocervical curettage (ECC) results. The selection of these variables was based on their clinical relevance and previously reported associations with residual disease in early-stage cervical cancer. All analyses were conducted using JMP software (version 14.0; SAS Institute Inc., Cary, NC, USA).

Ethical considerations

This study was conducted as an ancillary analysis of a previously approved clinical study by the Institutional Review Board of Kagoshima University Hospital (approval number: 20-K04). Written informed consent was obtained from all participants prior to enrollment. Clinical data were extracted retrospectively from the electronic medical records.

## Results

Patient characteristics

This study included 20 patients with stage IA2 cervical cancer who underwent modified radical or radical hysterectomy or trachelectomy following cervical conization at Kagoshima University Hospital between March 2014 and December 2023. The median age was 45 years (range: 25-63), and the median body mass index (BMI) was 22.2 kg/m² (range: 16.5-31.8). Histological subtypes included squamous cell carcinoma in 15 patients (75%) and adenocarcinoma in five patients (25%). LVSI was identified in seven patients (35%). The median depth of stromal invasion was 4 mm (range: 3.1-5.0 mm).

Regarding the surgical approach, laparoscopic surgery was performed in 12 patients (60%), and open surgery was performed in eight patients (40%). The surgical procedures included modified radical hysterectomy in 15 patients (75%), radical trachelectomy in four patients (20%), and modified radical trachelectomy in one patient (5%). No patients received adjuvant therapy (0%), and no cases of parametrial invasion were observed (0%). The median follow-up period was 45 months (range: 10-106 months), during which no recurrences were reported (Table 1). Although the median follow-up was 45 months, no recurrences or deaths were observed, and therefore, Kaplan-Meier survival analysis was not conducted, as it would not yield meaningful stratification.

**Table 1:** Clinicopathological characteristics BMI, body mass index; SCC, squamous cell carcinoma; LVSI, lymph vascular space invasion

Variable (N = 20)	Result
Median age (range), years	45 (25–63)
Median BMI (range), kg/m^2^	22.2 (16.5–31.8)
Final pathology	
SCC	15 (75%)
Adenocarcinoma	5 (25%)
LVSI	
No	13 (65%)
Yes	7 (35%)
Depth of invasion (range), mm	4 (3.1–5)
Parametrial invasion	
No	20 (100%)
Yes	0
Surgical approach	
Open	8 (40%)
Laparoscopy	12 (60%)
Type and radicality of surgery	
Modified radical hysterectomy	15 (75%)
Radical trachelectomy	4 (20%)
Modified radical trachelectomy	1 (5%)
Median operation time (range), min	230 (169–591)
Median blood loss (range), g	167 (5–1200)
Median length of hospital stays (range), days	7 (5–19)
Adjuvant therapy	
No	20 (100%)
Yes	0
Recurrence	
No	20 (100%)
Yes	0

SLN mapping outcomes

SLN mapping was successfully performed bilaterally in all patients (20/20, 100%). A total of 40 SLNs were identified. The most common locations were the external iliac region (21 nodes, 52.5%), followed by the obturator (15 nodes, 37.5%), internal iliac (three nodes, 7.5%), and common iliac (one node, 2.5%) regions. The median number of SLNs removed per patient was two. No SLN metastases were detected in any of the patients (Table 2). While these results are favorable, the complete absence of metastases may reflect selection bias, as the study cohort largely consisted of clinically low-risk cases. Caution is warranted in generalizing these findings to broader populations.

**Table 2:** Sentinel lymph node-related outcomes SLN: sentinel lymph node

Variable	Patients (N = 20)
SLN mapping	
Bilateral	20 (100%)
Unilateral	0
SLN locations (n = 40)	
External iliac	21 (52.5%)
Obturator	15 (37.5%)
Internal iliac	3 (15%)
Common iliac	1 (5%)
Median number of SLN removed	2
SLN metastasis	0

Univariate analysis of factors associated with residual tumor

Univariate analysis was conducted to identify clinicopathologic factors associated with residual tumor in the hysterectomy specimens following conization. All patients with a positive ECC (6/6, 100%) had residual disease, showing a statistically significant association (χ² = 8.57, p = 0.003). A positive endocervical margin was found in 12 patients, of whom eight (66.7%) had residual tumor, suggesting a trend but without statistical significance (χ² = 3.33, p = 0.06). No residual tumor was observed among patients with a positive ectocervical margin (0/2, 0%), and no significant association was found (χ² = 2.22, p = 0.14) (Table 3). These univariate results were not adjusted for multiple comparisons, given the exploratory intent and limited sample size, which we now explicitly acknowledge in the Discussion. Given the limited number of cases and events, these findings should be interpreted with caution.

**Table 3:** Univariate analysis of clinicopathologic predictors for residual tumor in hysterectomy specimens following cervical conization (n = 20) Statistical analysis: chi-square or Fisher's exact test for categorical variables. Statistical significance was set at p < 0.05. Residual tumor was defined as the presence of carcinoma or intraepithelial lesions in the final hysterectomy specimen.

Variable	No Residual Tumor (n = 10)	Residual Tumor (n = 10)	Test Statistic	p-value
Positive ectocervical margin	-	-	χ² = 2.22	0.14
Yes	2 (20%)	0	-	-
No	8 (80%)	10 (100%)	-	-
Positive endocervical margin	-	-	χ² = 3.33	0.06
Yes	4 (40%)	8 (80%)	-	-
No	6 (60%)	2 (20%)	-	-
Positive endocervical curettage	-	-	χ² = 8.57	0.003
Yes	0	6 (60%)	-	-
No	10 (100%)	4 (40%)	-	-

Multivariate logistic regression analysis

Multivariate logistic regression analysis revealed that a positive ECC was significantly associated with residual tumor in hysterectomy specimens (odds ratio (OR) = 8.38, 95% confidence interval (CI): 1.14-61.61, p = 0.0038). Histologic type also demonstrated a significant association; adenocarcinoma histology was associated with higher odds of residual tumor compared to squamous cell carcinoma (OR = 7.07, p = 0.0078), although the 95% CI could not be calculated due to sparse data and quasi-complete separation. In contrast, positive ectocervical margin (OR = 2.36 × 10⁻¹⁴, 95% CI: 1.77 × 10⁻³⁰ to 1.27 × 10³⁰, p = 0.0959) and positive endocervical margin (OR = 2.58 × 10³⁰, 95% CI: 0.0149-2.38 × 10³⁴, p = 0.464) were not significantly associated with residual tumor. The extremely wide CIs, particularly for the ectocervical margin, reflect model instability due to the small sample size and limited event count (10/20 with residual tumor). These limitations reduce the statistical power and suggest that the regression results should be interpreted with appropriate caution (Table 4).

**Table 4:** Multivariate logistic regression analysis of clinicopathologic factors associated with residual tumor in hysterectomy specimens after cervical conization (n = 20). Odds ratios (ORs), 95% confidence intervals (CIs), and p-values are presented. A p-value < 0.05 was considered statistically significant. ^†^ The 95% confidence interval for histologic type (adenocarcinoma vs. SCC) could not be calculated due to sparse data and quasi-complete separation. ^‡^ Extremely wide confidence intervals in certain variables (e.g., margin status) reflect limitations due to small sample size and data sparsity. SCC, squamous cell carcinoma

Variable	Odds Ratio (OR)	95% CI for OR (Lower–Upper)	p-value
Positive ectocervical margin	2.3624E–14	1.77 × 10⁻³⁰–1.27 × 10³⁰^‡^	0.0959
Positive endocervical margin	2.5813E+30	0.0149–2.38 × 10³⁴^‡^	0.464
Positive endocervical curettage	8.38	1.14–61.61	0.0038
Final pathology (adenocarcinoma vs. SCC)	7.07	Not calculable^†^	0.0078

## Discussion

This study aimed to evaluate the feasibility of conization with or without SLN biopsy as a fertility-sparing surgical option for patients with stage IA2 cervical cancer. Among 20 Japanese patients who underwent modified radical or radical hysterectomy/trachelectomy with SLN biopsy following conization, no recurrences were observed during a median follow-up period of 45 months. Multivariate analysis revealed that positive ECC and adenocarcinoma histology were independent predictors of residual tumor in the final hysterectomy specimen. These findings suggest that for selected IA2 patients with squamous cell carcinoma, negative endocervical margins, and negative ECC, conization ± SLN biopsy may serve as a less invasive alternative to radical trachelectomy for fertility preservation.

In recent years, the trend toward de-escalation of surgery for early-stage cervical cancer has gained momentum, with multiple studies reporting its safety and efficacy. Nica et al. [7] investigated conization combined with SLN biopsy in 44 patients with early-stage, low-risk cervical cancer (including IA2 cases) and reported no recurrences during a median follow-up period of 44 months. Similarly, Li et al. [10] evaluated 40 patients, including 21 with stage IA2 disease, who underwent conization and laparoscopic pelvic lymphadenectomy. Their study demonstrated an extremely low recurrence rate of 2.5%, along with favorable fertility outcomes.

The present study focused exclusively on stage IA2 disease and specifically assessed the clinical utility of pathological findings from conization specimens. Consistent with previous literature, positive ECC was significantly associated with residual tumor. This is supported by Diaz et al., who reported a 69% residual tumor rate when both ECC and endocervical margins were positive, compared to only 11% when both were negative [11].

Importantly, in our cohort, no residual tumor was found in any case where both ECC and the endocervical margin were negative. This supports previous findings by Baiocchi et al., who observed a low incidence of residual disease in patients with negative margins and smaller tumor volume [12]. These results suggest that when both ECC and endocervical margins are negative, it may be clinically appropriate to consider omitting radical surgery in favor of conization ± SLN biopsy, especially for fertility preservation, particularly in cases of squamous cell carcinoma. However, special consideration must be given to cases of adenocarcinoma. In cervical glandular lesions, skip lesions in the upper endocervix are not uncommon. Prior studies have reported that even in cases of adenocarcinoma in situ (AIS) with negative conization margins, residual disease in the uterus may still be present in up to 20% of cases [13]. In the current study, adenocarcinoma histology was independently associated with a significantly higher risk of residual tumor. Notably, one patient with adenocarcinoma had negative ECC and endocervical margins but was found to have residual AIS in the hysterectomy specimen. Thus, for adenocarcinoma, caution is warranted even when conization findings are favorable, and clinicians should consider tailoring surgical extent based on histology.

With respect to lymphatic evaluation, SLN mapping was successful bilaterally in all patients (100%), and no SLN metastases were observed. These findings support the feasibility and reliability of SLN biopsy as a staging tool in IA2 cervical cancer, particularly in the context of fertility-preserving strategies.

From the perspective of Japanese clinical practice, radical hysterectomy or trachelectomy remains the standard for IA2 cervical cancer, with conization alone rarely accepted due to concerns over residual disease and recurrence. However, our findings suggest that in highly selected patients who meet strict pathological criteria, conization ± SLN biopsy may be a viable and safe fertility-sparing option, aligning with the global trend toward individualized treatment.

However, several important limitations should be considered. First, this was a single-institution retrospective study with a small sample size, limiting the generalizability of the results. Second, selection bias is likely present, as our cohort only included patients who underwent further surgery after conization, most of whom presented with favorable clinical features. This may explain the complete absence of SLN metastases and recurrence, and therefore, the excellent SLN mapping results (100% success, 0% metastasis) may reflect a low-risk population. Third, despite the extended median follow-up period, no recurrences or deaths occurred, precluding the use of Kaplan-Meier survival analysis. Fourth, no corrections for multiple comparisons were applied in the univariate analysis, as the investigation was an exploratory study; however, this increases the risk of type I error. Fifth, the low number of events (10/20 with residual disease) and extremely wide CIs in multivariate regression models reflect limited statistical power and model instability, particularly regarding ectocervical margin status. These findings should, thus, be interpreted with caution. Finally, reproductive outcomes, such as pregnancy and live birth rates, were not assessed, which precludes an evaluation of the actual impact of this fertility-preserving approach.

## Conclusions

In conclusion, this study demonstrates that in patients with stage IA2 cervical cancer, positive ECC and adenocarcinoma histology are significantly associated with residual tumor in the final hysterectomy specimen. Importantly, no residual tumor was observed in patients with both negative ECC and negative endocervical margin, particularly among those with squamous cell carcinoma. These findings suggest that for highly selected patients, especially those with squamous histology, conization ± SLN biopsy may be cautiously considered as a fertility-sparing alternative to trachelectomy when both ECC and endocervical margins are negative. However, due to the small sample size and limited number of events, these results should be interpreted with caution. Further prospective studies with larger cohorts are necessary to validate the oncologic safety and long-term outcomes of this approach, particularly in patients with adenocarcinoma.

## References

[1] Arbyn M, Weiderpass E, Bruni L, de Sanjosé S, Saraiya M, Ferlay J, Bray F (2020). Estimates of incidence and mortality of cervical cancer in 2018: a worldwide analysis. Lancet Glob Health.

[2] Drolet M, Bénard É, Pérez N, Brisson M (2019). Population-level impact and herd effects following the introduction of human papillomavirus vaccination programmes: updated systematic review and meta-analysis. Lancet.

[3] Koh WJ, Abu-Rustum NR, Bean S (2019). Cervical cancer, version 3.2019, NCCN clinical practice guidelines in oncology. J Natl Compr Canc Netw.

[4] Seino M, Nagase S, Tokunaga H (2024). Japan Society of Gynecologic Oncology 2022 guidelines for uterine cervical neoplasm treatment. J Gynecol Oncol.

[5] Okugawa K, Yahata H, Ohgami T, Yasunaga M, Asanoma K, Kobayashi H, Kato K (2023). An update of oncologic and obstetric outcomes after abdominal trachelectomy using the FIGO 2018 staging system for cervical cancer: a single-institution retrospective analysis. J Gynecol Oncol.

[6] Wong AS, Li WH, Cheung TH (2016). Predictive factors for residual disease in hysterectomy specimens after conization in early-stage cervical cancer. Eur J Obstet Gynecol Reprod Biol.

[7] Nica A, Burnt A, Gauthier RJ (2021). Cervical conization and lymph node assessment for early stage low-risk cervical cancer. Int J Gynecol Cancer.

[8] Togami S, Tashiro H, Watanabe T (2024). Long-term outcomes of sentinel lymph node navigation surgery for early-stage cervical cancer. Int J Clin Oncol.

[9] Togami S, Fukuda M, Mizuno M, Yanazume S, Kobayashi H (2023). Efficacy and prognosis of robotic surgery with sentinel node navigation surgery in endometrial cancer. J Gynecol Oncol.

[10] Li X, Xia L, Chen X, Fu Y, Wu X (2020). Simple conization and pelvic lymphadenectomy in early-stage cervical cancer: a retrospective analysis and review of the literature. Gynecol Oncol.

[11] Diaz ES, Aoyama C, Baquing MA, Beavis A, Silva E, Holschneider C, Cass I (2014). Predictors of residual carcinoma or carcinoma-in-situ at hysterectomy following cervical conization with positive margins. Gynecol Oncol.

[12] Baiocchi G, Guimarães GC, Faloppa CC (2021). Predictive factors for residual disease after conization in cervical cancer. Ann Surg Oncol.

[13] Lea JS, Sheets EE, Duska LR (2002). Endocervical curettage at conization to predict residual cervical adenocarcinoma in situ. Gynecol Oncol.

